# Hypoxia Enhances the Proliferative Response of Macrophages to CSF-1 and Their Pro-Survival Response to TNF

**DOI:** 10.1371/journal.pone.0045853

**Published:** 2012-09-19

**Authors:** John A. Hamilton, Derek C. Lacey, Amanda Turner, Bernard de Kok, Jennifer Huynh, Glen M. Scholz

**Affiliations:** Arthritis and Inflammation Research Centre, University of Melbourne Department of Medicine, The Royal Melbourne Hospital Parkville, Victoria, Australia; Universidade Estadual de Campinas, Biology Institute, Brazil

## Abstract

In chronic inflammatory lesions there are increased numbers of macrophages with a possible contribution of enhanced survival/proliferation due, for example, to cytokine action; such lesions are often hypoxic. Prior studies have found that culture in low oxygen can promote monocyte/macrophage survival. We show here, using pharmacologic inhibitors, that the hypoxia-induced pro-survival response of macrophages exhibits a dependence on PI3-kinase and mTOR activities but surprisingly is suppressed by Akt and p38 MAPK activities. It was also found that in hypoxia at CSF-1 concentrations, which under normoxic conditions are suboptimal for macrophage proliferation, macrophages can proliferate more strongly with no evidence for alteration in CSF-1 receptor degradation kinetics. TNF promoted macrophage survival in normoxic conditions with an additive effect in hypoxia. The enhanced hypoxia-dependent survival and/or proliferation of macrophages in the presence of CSF-1 or TNF may contribute to their elevated numbers at a site of chronic inflammation.

## Introduction

In the absence of an appropriate stimulus, macrophages and neutrophils die by apoptosis thereby providing a mechanism for the resolution of an inflammatory response [1], [2]. Increased macrophage numbers at a site of chronic inflammation, such as the rheumatoid synovium, can correlate with poor disease progress [3], [4]. One contributing factor to these increased numbers, in addition to altered cell trafficking, could be their enhanced local survival/proliferation [5]. In the absence of sufficient signaling from the widely expressed growth factor, macrophage-colony stimulating factor (M-CSF or CSF-1), which is responsible for their development and maintenance in tissues [6], many macrophage lineage populations die by apoptosis [2]. CSF-1-dependent monocyte/macrophage survival is believed to be critically dependent on a pathway involving phosphatidylinositol 3-kinase (PI3-K) and Akt activities [7], [8], [9], [10].

Chronic inflammatory lesions rich in macrophages are often hypoxic due to reduced blood supply with this reduced tissue oxygen tension postulated to contribute to the pathology [11]. Tissue hypoxia can lead to cellular dysfunction and eventually cell death, and to maintain viability and activity cells must adapt to periods of hypoxia by adopting a strategy to maintain their ATP levels [11], [12]. Macrophages [13] and neutrophils [14] can adapt quite well to hypoxic conditions and a number of monocyte/macrophage changes have been reported such as altered phagocytosis, migration and gene expression [15], [16], [17], [18]. Like the response to human neutrophils [19], [20], but unlike that for many cell types including rodent macrophages and macrophage cell lines [21], [22], [23], [24], we recently showed for the first time that culture of CSF-1-starved murine bone marrow-derived macrophages (BMM) and human monocytes in low oxygen tension promoted cell survival by delaying apoptosis [25]. Glycolysis was also enhanced. It was proposed that this pro-survival effect on macrophage populations could contribute to their increased numbers at sites of chronic inflammation and in tumor lesions [25]. Changes in Akt activity and Bcl-2 family member expression in hypoxia-exposed BMM were noted but with no conclusions as to their role being able to be made; thus the signal transduction cascades mediating this pro-survival response to hypoxia remain to be elucidated.

TNF is a key pro-inflammatory cytokine and its blockade can be efficacious in chronic inflammatory/autoimmune diseases, such as rheumatoid arthritis. Its neutralization can lead to reduced macrophage numbers and increased apoptosis in the rheumatoid synovium with a correlation with efficacy being noted [5], [26], [27]. Like hypoxia [28], TNF can increase glucose uptake in macrophage-rich tissues [29] and human macrophages [28]; an additive effect was noted in hypoxia [28]. Enhanced glucose uptake by macrophages is a common response to a number of agents which promote macrophage survival [30], [31], [32], [33], [34]. In the literature dramatically opposed observations on BMM viability in response to TNF under normoxic conditions have been reported [35], [36], [37], [38].

We show here, using a series of specific pharmacologic inhibitors, that the hypoxia-induced pro-survival response of CSF-1-starved BMM exhibits a partial dependence on PI3-K and S6 kinase (S6K) activities but unexpectedly is enhanced by Akt and p38 MAPK inhibition. We also show that in hypoxia at CSF-1 concentrations, which under normoxic conditions are suboptimal for macrophage proliferation, macrophages can proliferate more strongly. We also found that TNF promoted BMM survival with an additive effect in hypoxia.

## Materials and Methods

### Ethics Statement

This work has been approved by the University of Melbourne Animal Ethics Committee.

### Reagents

Reagents used were as follows: recombinant human CSF-1 (Chiron), recombinant murine TNF (R&D Systems), propidium iodide (Sigma), wortmannin (Calbiochem), LY294002 (Merck), Akt VIII (Merck), rapamycin (Calbiochem), PD98059 (Merck), U0126 (Merck), SB203580 (Merck) and antibodies: anti-phosphotyrosine (4G10) (Millipore), monoclonal anti-CSF-1R (AFS98) (eBioscience), anti-CSF-1R and anti-Erk2 (Santa Cruz Biotech), anti-phospho-Tyr809 CSF-1R, anti-phospho-Thr202/Tyr204 Erk1/2, anti-phospho-Ser473 Akt, and anti-phospho-Tyr705 Stat3 (Cell Signaling Technology).

### Preparation of bone marrow-derived macrophages

Adherent bone marrow-derived macrophages (BMM) were generated from precursors in the presence of CSF-1 by a protocol similar to one previously described [25]. Briefly, bone marrow cells were isolated from the femurs of mice and cultured in RPMI 1640 media (Invitrogen), supplemented with penicillin (100 U/mL)/streptomycin (100 μg/mL), 20 mM HEPES (Invitrogen), and 10% heat-inactivated FBS in the presence of 5,000 U/mL of CSF-1 in Iwaki dishes. After a total of 5–7 days, adherent monolayers were harvested and the cells seeded into 6-well Iwaki plates (to allow for easier cell detachment at termination), again in the presence of the above growth medium, and incubated overnight under normoxic conditions (21% and 5% CO_2_) to allow for adherence. Following washing, the viable cell number was counted as the value at time  = 0.

### Hypoxic treatment

For hypoxia experiments, BMM from above were transferred from incubation overnight under normoxic conditions into a precalibrated hypoxic chamber (Coy Laboratories) with a 1% O_2_ and 5% CO_2_ flow [25].

### Viable cell number

Cell viability was measured using the trypan blue exclusion method. Cells were removed from Iwaki wells using either cell dissociation buffer (Sigma-Aldrich) as per the manufacturer's instructions or by incubating cells with PBS at 37°C for 20 min, followed by gentle pipetting to dislodge cells.

### Sub-G_0_/G_1_ DNA (apoptosis) assay

Briefly, dislodged BMM cells were resuspended in 50 µl of PBS, and 1 mL of ice-cold 70% ethanol was added. Cells were left overnight at 4°C and, on the following day, were pelleted, washed with PBS, and resuspended in 0.5 mL of a staining solution containing 69 µM propidium iodide and 5 µg/mL RNase A in 38 mM sodium citrate [39]. Stained cells were analyzed using a FACSCalibur (BD Biosciences) flow cytometer. All data analysis was conducted using CellQuest (version 3.1) (BD Biosciences).

### Cell lysis, SDS-PAGE, and Western blotting

Cells were lysed directly in tissue culture dishes with Nonidet P-40 lysis buffer (20 mM Tris-HC1 (pH 7.4), 150 mM NaCl, 1 mM EDTA, 1% Nonidet P-40, 10% glycerol, 1 mM sodium orthovanadate, 10 mM NaF, 10 mM β-glycerophosphate) containing Complete™ protease inhibitor cocktail (Roche) for 30 min on ice. Lysates were clarified by centrifugation at 15,000 rpm for 10 min at 4°C, and the protein concentrations measured using a protein assay kit (Bio-Rad). SDS-PAGE and Western blotting were performed by standard techniques [25].

## Results

### Effect of signaling pathway inhibitors on hypoxia-induced macrophage survival

**Figure 1:**
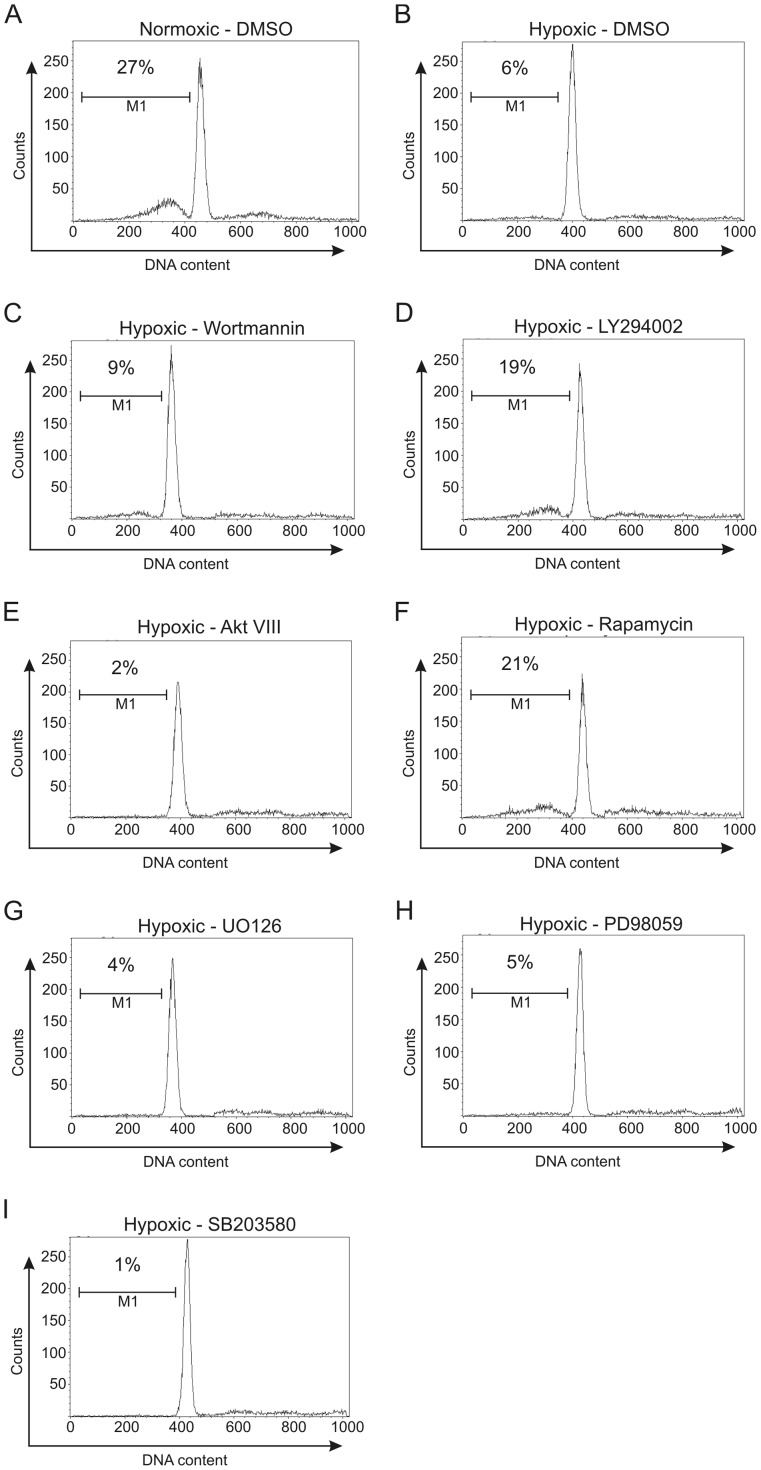
Propidium iodide analysis of apoptosis in hypoxic BMM treated with specific inhibitors. CSF-1-depleted BMM were incubated for 24 h in normoxic (A) and hypoxic (1% O2) conditions (B) and treated with DMSO vehicle. The hypoxic BMM were also treated with wortmannin (100 nM) (C), LY294002 (10 µM) (D), Akt VIII (10 µM) (E), rapamycin (60 nM) (F), U0126 (10 µM) (G), PD98059 (20 µM) (H), and SB203580 (10 µM) (I). DNA content that corresponds to apoptotic cells as defined by cells with <2N DNA (M1) is gated, and the percentage of total cells in M1 indicated. Data is representative of four separate experiments.

**Figure 2:**
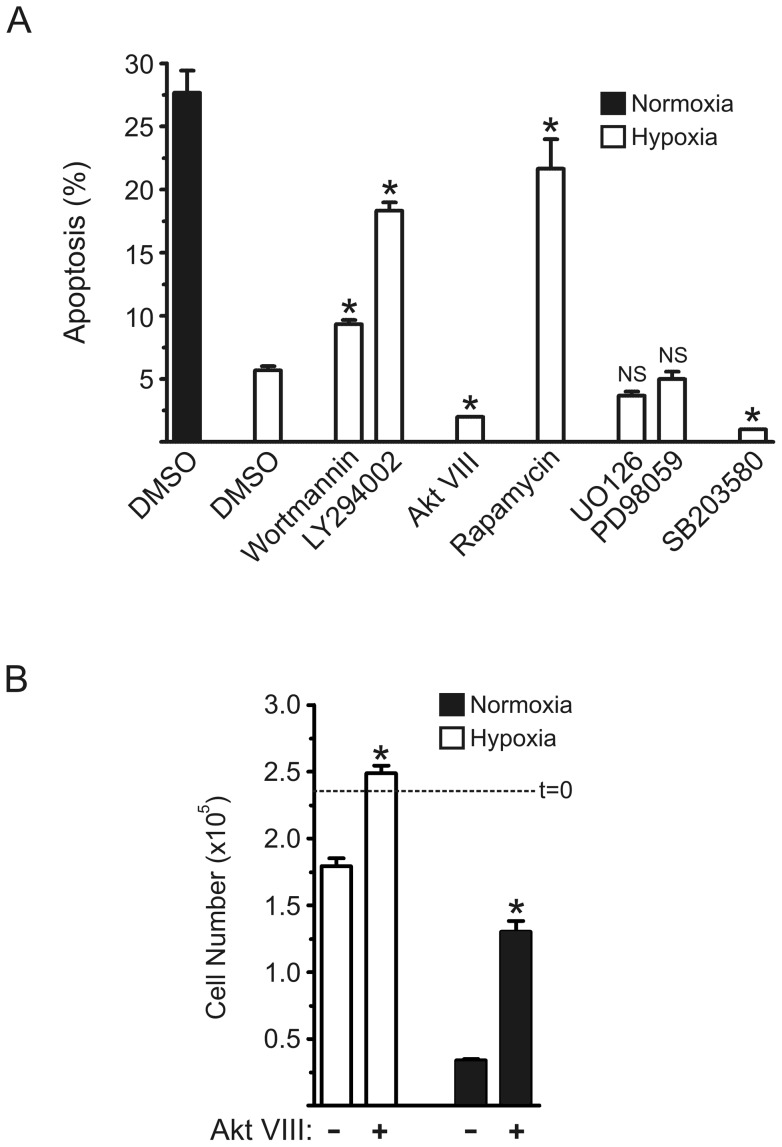
Apoptosis in hypoxic BMM in the presence of specific inhibitors. (A) The experimental protocol and analysis were as for the experiment in Figure 1. The data for the inhibitors are “clustered” based on pathway specificity; wortmannin and LY294002 (PI3-K), Akt VIII (Akt), rapamycin (mTOR), UO126 and PD98059 (MEK1/2), and SB203580 (p38 MAPK). Data are mean values (± SEM) of % apoptosis from triplicate cultures from a representative experiment (n = 5). *, p<0.05 (Student's t-test) vs hypoxia/DMSO group; NS, not significant. (B) BMM were depleted of CSF-1 and left untreated (DMSO vehicle) or treated in triplicate cultures with Akt VIII (10 µM), under hypoxic or normoxic conditions for 2 days. Viable cell number was counted (trypan blue exclusion); t = 0, initial cell number. Data are mean values (± SEM) from a representative experiment (n = 2); *, p<0.05 (Student's t-test) vs corresponding untreated group.

We showed before that the loss of viability of BMM upon CSF-1 withdrawal was reduced under hypoxic conditions [25]. In contrast to enhanced glucose uptake, no correlation was found for the enhanced survival with Akt activity and Bcl-2 family member expression. We therefore explored the pathways that may be involved in this pro-survival or anti-apoptotic effect of hypoxia by the addition of a number of pathway inhibitors. Using flow cytometry and the appearance of sub-G_0_/G_1_ DNA as an indicator of apoptosis in CSF-1-deprived BMM [39], when such CSF-1-starved BMM were placed under hypoxic conditions we observed less apoptosis – representative plots are provided in Figures 1A and 1B and mean values from replicate cultures given in Figure 2. Data for the effects of a series of pharmacologic inhibitors on this pro-survival effect of hypoxia are also presented in Figures 1C–I and Figure 2A. We see that the PI3-K inhibitors, wortmannin (Fig. 1C) and LY294002 (Fig. 1D), suppressed this pro-survival effect, measured over a 24 h period (Fig. 2A). Since PI3-K activity is upstream of Akt and S6K activities in stimulated BMM [40], [41] we tested the effect of their inhibitors, namely Akt VIII [33] and rapamycin [40], respectively (note: rapamycin prevents activation of S6K activity, for example, via its target, mTOR [41]). The latter inhibitor was capable of suppressing the anti-apoptotic response to hypoxia (Fig. 1F; Fig. 2A) but, surprisingly, there was less apoptosis in the presence of Akt VIII (Fig. 1E; Fig. 2A). Also there were more viable cells remaining when Akt VIII was added in hypoxia as well as under normoxic conditions for CSF-1-starved BMM (Fig. 2B). The MEK1/2 inhibitors, U0126 and PD98059, were ineffective in inhibiting the anti-apoptotic response to hypoxia (Figs. 1G and 1H; Fig. 2A); interestingly, there was less apoptosis in hypoxia-exposed BMM in the presence of the p38 MAPK inhibitor, SB203580 (Fig. 1I; Fig. 2A).

**Figure 3:**
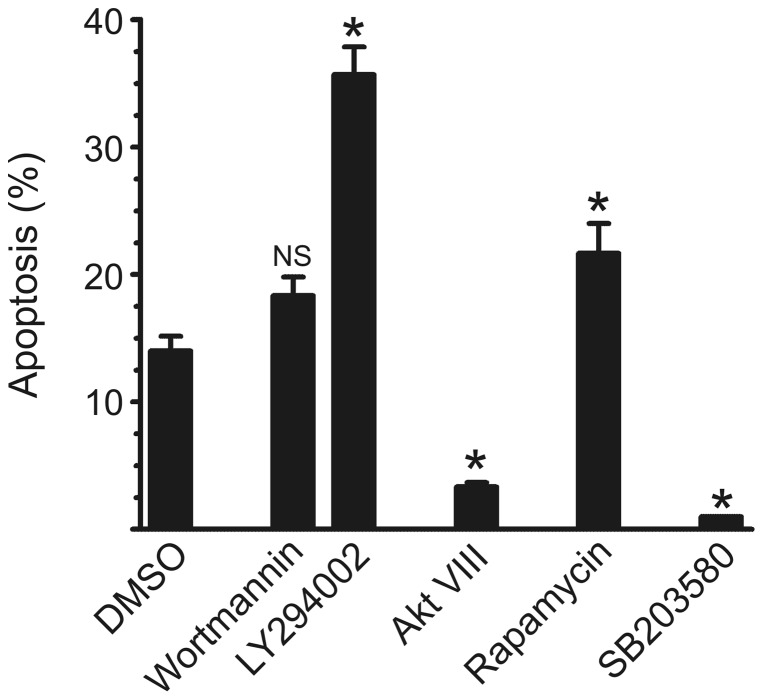
Apoptosis in CSF-1-treated BMM in the presence of specific inhibitors. CSF-1-depleted BMM were incubated in normoxic conditions for 24 h in the presence of CSF-1 (640 U/mL). The cells were also treated with DMSO (vehicle), wortmannin (100 nM), LY294002 (10 µM), Akt VIII (10 µM), rapamycin (30 nM) and SB203580 (10 µM). DNA content was measured as in Figure 1. Data for the inhibitors are “clustered” as in Figure 2. Data are mean values (±SEM) of % apoptosis from triplicate cultures from a representative experiment which was repeated at least three times. *, p<0.05 (Students' t test) vs DMSO group; NS, not significant.

Presumably the loss of viability in CSF-1-starved BMM [30] reflects the gradual decline in the CSF-1 signaling previously imparted to the BMM during their preparation and it could be that hypoxia prolongs this signaling in the absence of CSF-1. We therefore compared the effects of the same pathway inhibitors on the pro-survival action of CSF-1 under normoxic conditions. Using a low concentration of CSF-1 (640 U/mL) to induce BMM survival [30], [41], [42], [43], the effects of the same pathway inhibitors on the pro-survival effects of CSF-1 under normoxic conditions were similar to those noted above for CSF-1-depleted BMM in hypoxia (Fig. 3); in separate experiments, the MEK inhibitors, U0126 and PD 98059, gave at best only slight inhibition (data not shown). While completely inhibiting CSF-1-stimulated Akt activity in BMM, it was found recently that both wortmannin and Akt VIII also suppressed completely the CSF-1-stimulated glucose uptake [33]; constitutively active Akt also enhanced glucose uptake and the survival of CSF-1-depleted BMM. Based at least on this evidence and the data with PI3-K inhibitors, it would have been predicted that Akt VIII would suppress CSF-1-induced BMM survival. However, unlike LY294002, as for hypoxia exposure, there was again even less apoptosis in CSF-1-treated BMM in the presence of Akt VIII (Fig. 3).

### Effect of hypoxia on macrophage proliferation in the presence of CSF-1

#### Cell proliferation

**Figure 4:**
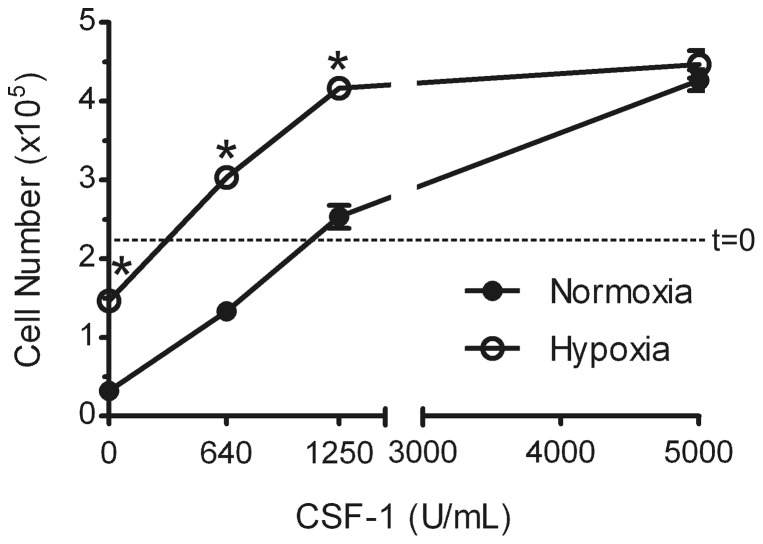
Hypoxia promotes BMM proliferation in the presence of suboptimal CSF-1 concentrations. BMM were depleted of CSF-1 and left untreated or treated in triplicate cultures with increasing CSF-1 concentrations under hypoxic or normoxic conditions for 2 days. Viable cell number was counted; t = 0, initial cell number. Data are mean values (± SEM) from a representative experiment (n = 5). *, p<0.05 (Student's t-test) vs corresponding normoxia group.

Macrophages in tissues are likely to be exposed to the ubiquitously expressed CSF-1 [44]. We showed previously [25] that when BMM were exposed to hypoxic conditions in the presence of low or pro-survival concentrations of CSF-1, the pro-survival response to such concentrations was also enhanced. At the high CSF-1 concentrations used for their generation from bone marrow precursors, the adherent BMM themselves proliferate [40], [41], [42]. We therefore tested whether there might be an increased proliferative response under hypoxic conditions to CSF-1 concentrations which normally give a suboptimal proliferative response. We see for the experiment whose data are shown in Figure 4 that the hypoxic BMM did in fact show an enhanced proliferation when compared with their normoxic counterparts at such a concentration (1250 U/mL); also in the experiment shown proliferation was observed in hypoxia at 640 U/mL which resulted only in some maintenance of cell number under normoxic conditions.

#### CSF-1 receptor signaling

**Figure 5:**
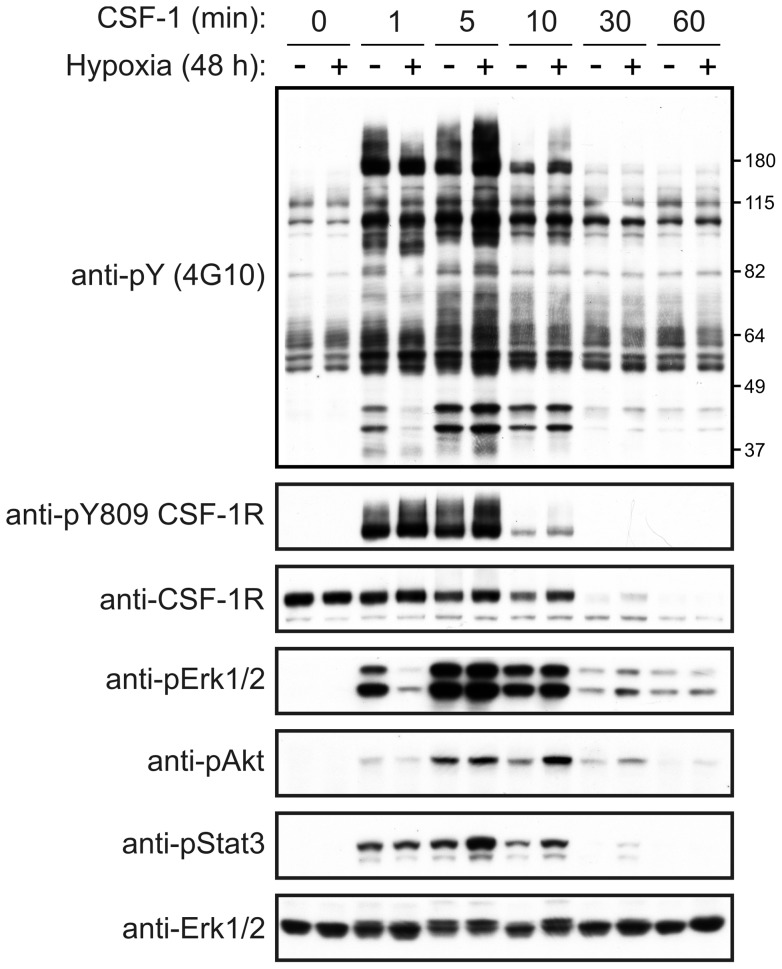
Hypoxia and CSF-1 receptor signaling. BMM were cultured under hypoxic and normoxic conditions for 48 h, with CSF-1 having been removed during the final 16 h. The BMM were subsequently stimulated with CSF-1 and lysed at the indicated time points. Aliquots of the cell lysates were subjected to Western blotting with anti-pY (4G10), anti-phospho-Y809 CSF-1R, anti-CSF-1R, anti-phospho-Erk1/2, anti-phospho-Akt, and anti-phospho-Stat3 antibodies. Membranes were also probed with an anti-Erk2 antibody to determine protein loading levels. Data are representative of three independent experiments.

Given the findings above that hypoxia can potentiate the mitogenic effects of CSF-1 on BMM, we investigated the consequences of hypoxia on signaling by the CSF-1 receptor. The magnitude and duration of CSF-1 receptor autophosphorylation at Tyr809 (within the catalytic loop of the kinase domain [45]) appeared unaffected by hypoxia as did the kinetics of the CSF-1-induced degradation of its receptor (Fig. 5). Notably, CSF-1-induced Erk1/2 activation was delayed in hypoxia, but with no obvious change in Akt activation. In the case of Stat3, there appeared to be a slight but transient increase in its activation in response to CSF-1 stimulation in hypoxia (Fig. 5).

### Effect of hypoxia on macrophage survival in the presence of TNF

**Figure 6:**
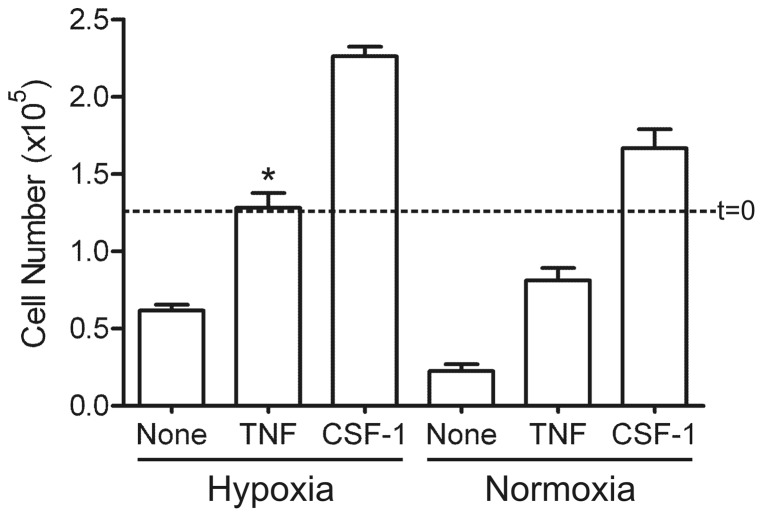
Enhanced BMM survival by TNF in normoxic and hypoxic conditions. BMM were depleted of CSF-1 and left untreated or treated with TNF (10 ng/mL) or CSF-1 (5000 U/mL), under hypoxic or normoxic conditions for 2 days. Viable cell number was counted; t = 0, initial cell number. Data are mean values (± SEM) from triplicate cultures from a representative experiment (n = 5). *, p<0.05 (Student's t-test) vs. TNF-treated group in normoxia.

Another cytokine to which macrophages in an inflammatory and hypoxic environment are likely to be exposed is TNF [11], [26]. Like hypoxia [25], [28], TNF can stimulate glucose uptake in macrophages [28]; an additive effect was noted under hypoxic conditions. Since enhanced glucose uptake has been implicated in the pro-survival effects on macrophages of a number of stimuli [30], [31], [32], [33], [34], we considered that there might also be an additive effect on macrophage survival between TNF and hypoxia. We first measured the viability response of CSF-1-starved BMM to TNF under normoxic conditions. As observed in Figure 6, BMM exhibited a pro-survival, but not a mitogenic, response to TNF – the data shown are for an optimal concentration (10 ng/mL). One possibility is that endogenous CSF-1 is being stimulated by the TNF and thereby mediating its action. This mechanism seems unlikely, however, since a similar TNF dose response for survival was noted for BMM from *Csf1^op^/Csf1^op^* mice (i.e. mice with an inactivating CSF-1 mutation [44]); also neutralizing anti-CSF-1 receptor monoclonal antibody (AFS-98), while suppressing the pro-survival effect of CSF-1 as expected, failed to block that due to TNF (data not shown). We drew a similar conclusion before for the pro-survival effect of hypoxia by using *Csf1^op^/Csf1^op^* BMM [25].

There was an additive pro-survival affect when BMM were cultured in hypoxia and in optimal TNF concentration (Fig. 6); a similar effect was noted at sub-optimal TNF concentrations (data not shown). It can be seen that, unlike the response to CSF-1 (Fig. 4), no increase in cell number, relative to the starting value, was found for the TNF-treated cells in hypoxia. Thus under both normoxic and hypoxic conditions, in our hands TNF, unlike CSF-1, was a pro-survival factor for BMM but not a mitogen.

## Discussion

**Figure 7:**
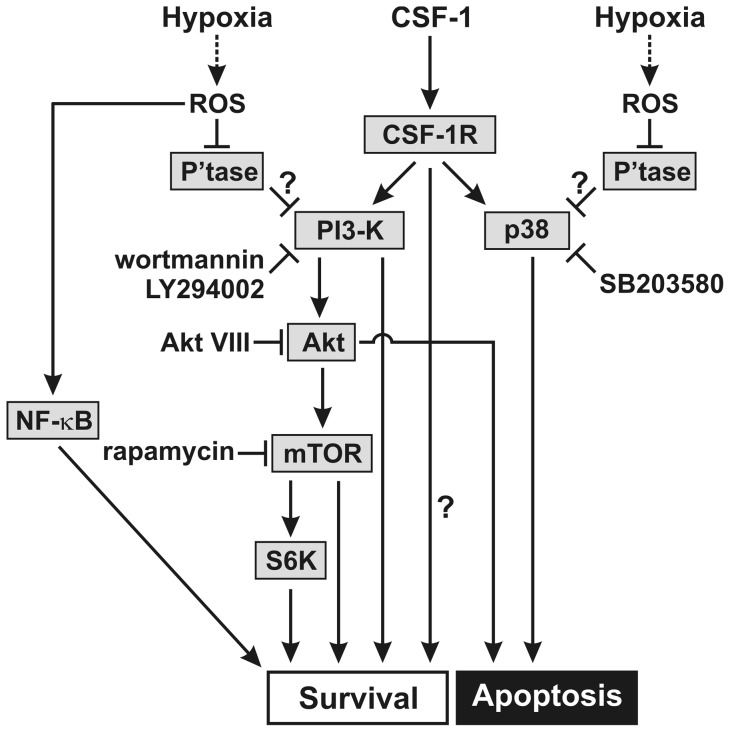
Pathways regulating hypoxia- and CSF-1-induced macrophage survival. CSF-1-depleted BMM, which die by apoptosis, were exposed to hypoxia and a “pro-survival” CSF-1 concentration. The working model illustrates the putative signaling pathways that regulate hypoxia- and CSF-1-induced macrophage survival based on inhibitor effects. In the presence of both stimuli, PI3-K inhibitors (wortmannin and LY294002) and the mTOR inhibitor (rapamycin) suppress macrophage survival, whereas the Akt inhibitor (Akt VIII) and the p38 MAPK inhibitor (SB203580) suppress apoptosis. It is proposed that hypoxia may regulate macrophage survival independently of CSF-1 via reactive oxygen species (ROS)-mediated NF-κB activation. Hypoxia may also modulate CSF-1-mediated macrophage survival/apoptosis again via ROS, which antagonize the phosphatases negatively regulating PI3-K and p38 MAPK activity, respectively. Possible other CSF-1-dependent pro-survival pathways are indicated (?). The Akt- and p38 MAPK-dependent signaling pathways putatively involved in regulating macrophage apoptosis are also shown.

The above findings extend our prior ones showing that hypoxia, in contrast to its effects on most other cell types [14], can prolong CSF-1-starved BMM and human monocyte survival with implications for the increased macrophage numbers in hypoxic inflammatory lesions [25]. Using pharmacologic inhibitors, it would appear that there is some dependence on a pathway(s) involving PI3-K activity and mTOR activity (possibly via S6K), but a lack of dependence on Erk1/2 activity. Surprisingly, inhibition of both Akt and p38 MAPK activities led to even less apoptosis than in hypoxia alone. By similar inhibitor approach to ours, direct hypoxia-mediated human neutrophil survival was found to be independent of the PI3-K pathway [20]. The pattern of the survival response in the presence of the various inhibitors was similar for hypoxic CSF-1-starved BMM, on the one hand, and for CSF-1-treated BMM on the other. This would tend to suggest that there are at least some common elements (i.e. convergence) to the respective signaling pathways through which hypoxia and CSF-1 exert their pro-survival effects on macrophages (Fig. 7). Given that intracellular levels of reactive oxygen species (ROS) can become elevated under hypoxic conditions, including in macrophages [46], the pro-survival effects of hypoxia are potentially mediated, at least in part, via the actions of ROS on CSF-1 signaling. For instance, ROS have recently been shown to enhance CSF-1-induced PI3-K signaling in BMM through the inhibitory effects of ROS on the phosphatase, SHP-1 [47]. Thus, PI3-K could represent a convergence point for hypoxia- and CSF-1-mediated survival signaling pathways in macrophages (Fig. 7). Hypoxia could also promote BMM survival independently of CSF-1 signaling through the activation of NF-κB by ROS [48], [49] (Fig. 7). Another possible explanation is that endogenous CSF-1 is mediating the pro-survival effect of hypoxia as discussed before [25]. However, this indirect mechanism would not appear to be operating since we found previously [25] that hypoxia promoted survival of BMM from *Csf1^op^/Csf1^op^* mice with an inactivating CSF-1 mutation.

Since expression of constitutively active Akt partially enhanced the survival of CSF-1-depleted BMM [33] and human monocytes [10], and given the prior literature implicating a PI3-K/Akt pathway in CSF-1-mediated monocyte/macrophage survival [7], [9], [10], [50] and glucose uptake [33], it was assumed that the inhibitor, Akt VIII, would suppress such survival and also that enhanced by hypoxia, i.e. opposite to what we found above. In this context, however, it was recently reported that Akt activity was not required for BMM survival; instead Akt was shown to be required for the differentiation of BMM into osteoclasts [51]. In the same study mTOR was shown to have a critical role in mediating BMM survival [51], a finding consistent with our own data here with rapamycin. As judged by the effects of LY294002, we found some dependence on PI3-K activity for the pro-survival response to CSF-1. The prior studies with CSF-1-treated monocytes/macrophages examining the role of a PI3-K/Akt pathway [7], [9], [10], [50] did not employ Akt inhibition; further blockade/depletion studies are required to clarify whether Akt has in fact a suppressive role in both hypoxia- and CSF-1-mediated macrophage lineage survival, as suggested by our results with Akt VIII Fig. 7). Additional strategies are also needed to confirm the potentiation by p38 MAPK inhibition of hypoxia-and CSF-1-mediated survival found above with its implication of a pro-apoptotic effect of p38 MAPK under the particular cell conditions (Fig. 7). Others have also found that p38 MAPK inhibition did not suppress CSF-1-induced BMM survival [9]. Whether there is a link between Akt and p38 MAPK pathways in our studies, as has been reported in other cell types [52], [53], is unknown.

We also showed above in hypoxia that CSF-1 concentrations, which are suboptimal for BMM proliferation under normoxic conditions, BMM can in fact proliferate more strongly at least over the time scale examined. These data for hypoxia are similar to what we found with other stimuli, such as low concentrations of oxidized LDL and particulate adjuvants [43], [54], [55], and extend our previous observations on the enhanced survival of BMM to CSF-1 in hypoxia [25]. Thus hypoxia can be added to the list of stimuli [55] which can determine the minimum concentration of CSF-1 required to give a mitogenic response in certain macrophage lineage populations, i.e. determine whether a particular (low) CSF-1 concentration can induce proliferation or just survival. Since macrophages in vivo are likely to be in the presence of CSF-1 in tissues [44], the enhanced hypoxia-dependent survival/proliferation of macrophages in the presence of sub-optimal CSF-1 concentrations could also be relevant for their increased numbers in chronic inflammatory lesions, for example, the rheumatoid synovium [5], [56].

It was recently reported that the rate at which the EGF receptor is internalized into early endosomes, and subsequently degraded in the lysosomal compartment, is reduced in hypoxia [57]. This finding led the authors to suggest that delayed endocytic trafficking of the EGF receptor in hypoxia may ultimately drive tumor cell survival through more sustained EGF receptor-mediated signaling. Following its CSF-1-induced internalization, the CSF-1 receptor is trafficked via the endocytic system to lysosomes where it is degraded; this is believed to represent a key mechanism for preventing on-going signaling by the CSF-1 receptor [45]. A slower rate of CSF-1 receptor endocytosis in hypoxia could therefore potentially explain the enhanced mitogenic effects of CSF-1 on BMM under those conditions. However, there was no evidence for a hypoxia-mediated alteration in CSF-1R degradation or autophosphorylation. The significance of the slight delay in CSF-1-induced Erk1/2 activation in hypoxia is unknown. Thus it is unknown how the signaling output arising from the stimulation of macrophages in hypoxia with a CSF-1 concentration that is typically sub-optimal for macrophage survival and/or proliferation becomes equivalent to that triggered under normoxic conditions by an optimal CSF-1 concentration.

In agreement with the literature for human monocytes [58] and for BMM in certain studies, but for some reason not in others [35], [36], [37], we observed that TNF also promoted BMM survival. As proposed for CSF-1, this pro-survival response may contribute to the increased numbers of macrophages in the tissues of chronic inflammatory diseases [5], [56]. In this connection, in the rheumatoid synovium macrophage numbers are reduced following TNF blockade [26], [27] and reduced macrophage survival has been suggested as one mechanism [5]. Under hypoxic conditions the anti-apoptotic effect of an optimal TNF concentration was enhanced even further. This additive effect of TNF and hypoxia could be related to their additive effects on BMM glucose uptake noted [28]. Our data are consistent with the possibility that TNF can prolong macrophage survival particularly in a hypoxic environment, such as a site of an inflammatory reaction, and perhaps have relevance for the proposed interdependence of the innate and hypoxic responses to tissue injury and infection [59]. Unlike CSF-1, TNF was not a mitogen for BMM under either normoxic or hypoxic conditions. The signal transduction pathways governing the TNF pro-survival effect noted above were not examined but have been by others [37], [38]; in these studies, the PI3/Akt pathway, Akt activity and expression levels of Bcl-X_L_ and Bcl-2 did not correlate with pro- or anti-apoptotic responses although there was evidence for NF-κB-dependence. It is likely that the signaling pathways governing TNF-mediated survival in macrophages will show significant differences from those utilized by CSF-1 given their vastly different receptors.

## References

[1] Haslett C, Savill JS, Whyte MK, Stern M, Dransfield I, et al (1994) Granulocyte apoptosis and the control of inflammation. Philos Trans R Soc Lond B Biol Sci 345: 327–333. 7846130 10.1098/rstb.1994.0113

[2] Pope RM (2002) Apoptosis as a therapeutic tool in rheumatoid arthritis. Nat Rev Immunol 2: 527–535. 12094227 10.1038/nri846

[3] Tak PP, Bresnihan B (2000) The pathogenesis and prevention of joint damage in rheumatoid arthritis: advances from synovial biopsy and tissue analysis. Arthritis Rheum 43: 2619–2633. 11145019 10.1002/1529-0131(200012)43:12<2619::AID-ANR1>3.0.CO;2-V

[4] Pollard JW (2004) Tumour-educated macrophages promote tumour progression and metastasis. Nat Rev Cancer 4: 71–78. 14708027 10.1038/nrc1256

[5] Hamilton JA, Tak PP (2009) The dynamics of macrophage lineage populations in inflammatory and autoimmune diseases. Arthritis Rheum 60: 1210–1221. 19404968 10.1002/art.24505

[6] Tushinski RJ, Oliver IT, Guilbert LJ, Tynan PW, Warner JR, et al (1982) Survival of mononuclear phagocytes depends on a lineage-specific growth factor that the differentiated cells selectively destroy. Cell 28: 71–81. 6978185 10.1016/0092-8674(82)90376-2

[7] Kelley TW, Graham MM, Doseff AI, Pomerantz RW, Lau SM, et al (1999) Macrophage colony-stimulating factor promotes cell survival through Akt/protein kinase B. J Biol Chem. 274: 26393–26398. 10.1074/jbc.274.37.26393 10473597

[8] Murray JT, Craggs G, Wilson L, Kellie S (2000) Mechanism of phosphatidylinositol 3-kinase-dependent increases in BAC1.2F5 macrophage-like cell density in response to M-CSF: phosphatidylinositol 3-kinase inhibitors increase the rate of apoptosis rather than inhibit DNA synthesis. Inflamm Res 49: 610–618. 11131301 10.1007/s000110050638

[9] Comalada M, Xaus J, Sanchez E, Valledor AF, Celada A (2004) Macrophage colony-stimulating factor-, granulocyte-macrophage colony-stimulating factor-, or IL-3-dependent survival of macrophages, but not proliferation, requires the expression of p21(Waf1) through the phosphatidylinositol 3-kinase/Akt pathway. Eur J Immunol 34: 2257–2267. 15259023 10.1002/eji.200425110

[10] Wang Y, Zeigler MM, Lam GK, Hunter MG, Eubank TD, et al (2007) The role of the NADPH oxidase complex, p38 MAPK, and Akt in regulating human monocyte/macrophage survival. Am J Respir Cell Mol Biol 36: 68–77. 16931806 10.1165/rcmb.2006-0165OC PMC1899309

[11] Muz B, Khan MN, Kiriakidis S, Paleolog EM (2009) Hypoxia. The role of hypoxia and HIF-dependent signalling events in rheumatoid arthritis. Arthritis Res Ther 11: 201. 19222864 10.1186/ar2568 PMC2688222

[12] Mazumder B, Li X, Barik S (2010) Translation control: a multifaceted regulator of inflammatory response. J Immunol 184: 3311–3319. 20304832 10.4049/jimmunol.0903778 PMC2860598

[13] Cramer T, Yamanishi Y, Clausen BE, Forster I, Pawlinski R, et al (2003) HIF-1alpha is essential for myeloid cell-mediated inflammation. Cell 112: 645–657. 12628185 10.1016/s0092-8674(03)00154-5 PMC4480774

[14] Walmsley SR, Chilvers ER, Whyte MK (2009) Hypoxia. Hypoxia, hypoxia inducible factor and myeloid cell function. Arthritis Res Ther 11: 219. 19435530 10.1186/ar2632 PMC2688173

[15] Negus RP, Turner L, Burke F, Balkwill FR (1998) Hypoxia down-regulates MCP-1 expression: implications for macrophage distribution in tumors. J Leukoc Biol 63: 758–765. 9620670 10.1002/jlb.63.6.758

[16] Lewis JS, Lee JA, Underwood JC, Harris AL, Lewis CE (1999) Macrophage responses to hypoxia: relevance to disease mechanisms. J Leukoc Biol 66: 889–900. 10614769 10.1002/jlb.66.6.889

[17] Murdoch C, Muthana M, Lewis CE (2005) Hypoxia regulates macrophage functions in inflammation. J Immunol 175: 6257–6263. 16272275 10.4049/jimmunol.175.10.6257

[18] Elbarghati L, Murdoch C, Lewis CE (2008) Effects of hypoxia on transcription factor expression in human monocytes and macrophages. Immunobiology 213: 899–908. 18926304 10.1016/j.imbio.2008.07.016

[19] Hannah S, Mecklenburgh K, Rahman I, Bellingan GJ, Greening A, et al (1995) Hypoxia prolongs neutrophil survival in vitro. FEBS Lett 372: 233–237. 7556675 10.1016/0014-5793(95)00986-j

[20] Walmsley SR, Print C, Farahi N, Peyssonnaux C, Johnson RS, et al (2005) Hypoxia-induced neutrophil survival is mediated by HIF-1alpha-dependent NF-kappaB activity. J Exp Med 201: 105–115. 15630139 10.1084/jem.20040624 PMC2212759

[21] Rymsa B, Becker HD, Lauchart W, de Groot H (1990) Hypoxia/reoxygenation injury in liver: Kupffer cells are much more vulnerable to reoxygenation than to hypoxia. Res Commun Chem Pathol Pharmacol 68: 263–266. 2353135

[22] Yun JK, McCormick TS, Villabona C, Judware RR, Espinosa MB, et al (1997) Inflammatory mediators are perpetuated in macrophages resistant to apoptosis induced by hypoxia. Proc Natl Acad Sci U S A 94: 13903–13908. 9391125 10.1073/pnas.94.25.13903 PMC28405

[23] Fong CC, Zhang Q, Shi YF, Wu RS, Fong WF, et al (2007) Effect of hypoxia on RAW264.7 macrophages apoptosis and signaling. Toxicology 235: 52–61. 17416447 10.1016/j.tox.2007.03.006

[24] Degrossoli A, Giorgio S (2007) Functional alterations in macrophages after hypoxia selection. Exp Biol Med (Maywood) 232: 88–95. 17202589

[25] Roiniotis J, Dinh H, Masendycz P, Turner A, Elsegood CL, et al (2009) Hypoxia prolongs monocyte/macrophage survival and enhanced glycolysis is associated with their maturation under aerobic conditions. J Immunol 182: 7974–7981. 19494322 10.4049/jimmunol.0804216

[26] Smeets TJ, Kraan MC, van Loon ME, Tak PP (2003) Tumor necrosis factor alpha blockade reduces the synovial cell infiltrate early after initiation of treatment, but apparently not by induction of apoptosis in synovial tissue. Arthritis Rheum 48: 2155–2162. 12905468 10.1002/art.11098

[27] Catrina AI, Trollmo C, af Klint E, Engstrom M, Lampa J, et al (2005) Evidence that anti-tumor necrosis factor therapy with both etanercept and infliximab induces apoptosis in macrophages, but not lymphocytes, in rheumatoid arthritis joints: extended report. Arthritis Rheum 52: 61–72. 15641091 10.1002/art.20764

[28] Matsui T, Nakata N, Nagai S, Nakatani A, Takahashi M, et al (2009) Inflammatory cytokines and hypoxia contribute to 18F-FDG uptake by cells involved in pannus formation in rheumatoid arthritis. J Nucl Med 50: 920–926. 19443596 10.2967/jnumed.108.060103

[29] Meszaros K, Lang CH, Bagby GJ, Spitzer JJ (1987) Tumor necrosis factor increases in vivo glucose utilization of macrophage-rich tissues. Biochem Biophys Res Commun 149: 1–6. 3689407 10.1016/0006-291x(87)91596-8

[30] Hamilton JA, Vairo G, Lingelbach SR (1986) CSF-1 stimulates glucose uptake in murine bone marrow-derived macrophages. Biochem Biophys Res Commun 138: 445–454. 3488736 10.1016/0006-291x(86)90301-3

[31] Hamilton JA, Vairo G, Lingelbach SR (1988) Activation and proliferation signals in murine macrophages: stimulation of glucose uptake by hemopoietic growth factors and other agents. J Cell Physiol 134: 405–412. 2832422 10.1002/jcp.1041340311

[32] Elsegood CL, Chang M, Jessup W, Scholz GM, Hamilton JA (2009) Glucose metabolism is required for oxidized LDL-induced macrophage survival: role of PI3K and Bcl-2 family proteins. Arterioscler Thromb Vasc Biol 29: 1283–1289. 19667115 10.1161/ATVBAHA.108.180778

[33] Chang M, Hamilton JA, Scholz GM, Masendycz P, Macaulay SL, et al (2009) Phosphatidylinostitol-3 kinase and phospholipase C enhance CSF-1-dependent macrophage survival by controlling glucose uptake. Cell Signal 21: 1361–1369. 19376223 10.1016/j.cellsig.2009.04.003

[34] Chang M, Hamilton JA, Scholz GM, Elsegood CL (2009) Glycolytic control of adjuvant-induced macrophage survival: role of PI3K, MEK1/2, and Bcl-2. J Leukoc Biol 85: 947–956. 19270084 10.1189/jlb.0908522

[35] Xaus J, Comalada M, Valledor AF, Lloberas J, Lopez-Soriano F, et al (2000) LPS induces apoptosis in macrophages mostly through the autocrine production of TNF-alpha. Blood 95: 3823–3831. 10845916

[36] Comalada M, Xaus J, Valledor AF, Lopez-Lopez C, Pennington DJ, et al (2003) PKC epsilon is involved in JNK activation that mediates LPS-induced TNF-alpha, which induces apoptosis in macrophages. Am J Physiol Cell Physiol 285: C1235–1245. 12867362 10.1152/ajpcell.00228.2003

[37] Lombardo E, Alvarez-Barrientos A, Maroto B, Bosca L, Knaus UG (2007) TLR4-mediated survival of macrophages is MyD88 dependent and requires TNF-alpha autocrine signalling. J Immunol 178: 3731–3739. 17339471 10.4049/jimmunol.178.6.3731

[38] Lo SZ, Steer JH, Joyce DA (2011) TNF-alpha renders macrophages resistant to a range of cancer chemotherapeutic agents through NF-kappaB-mediated antagonism of apoptosis signalling. Cancer Lett 307: 80–92. 21482450 10.1016/j.canlet.2011.03.020

[39] Sester DP, Brion K, Trieu A, Goodridge HS, Roberts TL, et al (2006) CpG DNA activates survival in murine macrophages through TLR9 and the phosphatidylinositol 3-kinase-Akt pathway. J Immunol 177: 4473–4480. 16982883 10.4049/jimmunol.177.7.4473

[40] Hamilton JA, Byrne R, Whitty G, Vadiveloo PK, Marmy N, et al (1998) Effects of wortmannin and rapamycin on CSF-1-mediated responses in macrophages. Int J Biochem Cell Biol 30: 271–283. 9608681 10.1016/s1357-2725(97)00111-8

[41] Hamilton JA, Byrne R, Jessup W, Kanagasundaram V, Whitty G (2001) Comparison of macrophage responses to oxidized low-density lipoprotein and macrophage colony-stimulating factor (M-CSF or CSF-1). Biochem J 354: 179–187. 11171093 10.1042/0264-6021:3540179 PMC1221642

[42] Vairo G, Hamilton JA (1985) CSF-1 stimulates Na+K+-ATPase mediated 86Rb+ uptake in mouse bone marrow-derived macrophages. Biochem Biophys Res Commun 132: 430–437. 2998364 10.1016/0006-291x(85)91040-x

[43] Hamilton JA, Myers D, Jessup W, Cochrane F, Byrne R, et al (1999) Oxidized LDL can induce macrophage survival, DNA synthesis, and enhanced proliferative response to CSF-1 and GM-CSF. Arterioscler Thromb Vasc Biol 19: 98–105. 9888871 10.1161/01.atv.19.1.98

[44] Cecchini MG, Dominguez MG, Mocci S, Wetterwald A, Felix R, et al (1994) Role of colony stimulating factor-1 in the establishment and regulation of tissue macrophages during postnatal development of the mouse. Development 120: 1357–1372. 8050349 10.1242/dev.120.6.1357

[45] Pixley FJ, Stanley ER (2004) CSF-1 regulation of the wandering macrophage: complexity in action. Trends Cell Biol 14: 628–638. 15519852 10.1016/j.tcb.2004.09.016

[46] Chandel NS, Trzyna WC, McClintock DS, Schumacker PT (2000) Role of oxidants in NF-kappa B activation and TNF-alpha gene transcription induced by hypoxia and endotoxin. J Immunol 165: 1013–1021. 10878378 10.4049/jimmunol.165.2.1013

[47] Choi HK, Kim TH, Jhon GJ, Lee SY (2011) Reactive oxygen species regulate M-CSF-induced monocyte/macrophage proliferation through SHP1 oxidation. Cell Signal 23: 1633–1639. 21664461 10.1016/j.cellsig.2011.05.017

[48] Morgan MJ, Liu ZG (2011) Crosstalk of reactive oxygen species and NF-kappaB signaling. Cell Res 21: 103–115. 21187859 10.1038/cr.2010.178 PMC3193400

[49] Bubici C, Papa S, Dean K, Franzoso G (2006) Mutual cross-talk between reactive oxygen species and nuclear factor-kappa B: molecular basis and biological significance. Oncogene 25: 6731–6748. 17072325 10.1038/sj.onc.1209936

[50] Murray J, Wilson L, Kellie S (2000) Phosphatidylinositol-3′ kinase-dependent vesicle formation in macrophages in response to macrophage colony stimulating factor. J Cell Sci 113 Pt 2: 337–348. 10.1242/jcs.113.2.337 10633084

[51] Sugatani T, Hruska KA (2005) Akt1/Akt2 and mammalian target of rapamycin/Bim play critical roles in osteoclast differentiation and survival, respectively, whereas Akt is dispensable for cell survival in isolated osteoclast precursors. J Biol Chem 280: 3583–3589. 15545269 10.1074/jbc.M410480200

[52] Kim HJ, Oh JE, Kim SW, Chun YJ, Kim MY (2008) Ceramide induces p38 MAPK-dependent apoptosis and Bax translocation via inhibition of Akt in HL-60 cells. Cancer Lett 260: 88–95. 18054155 10.1016/j.canlet.2007.10.030

[53] Rane MJ, Coxon PY, Powell DW, Webster R, Klein JB, et al (2001) p38 Kinase-dependent MAPKAPK-2 activation functions as 3-phosphoinositide-dependent kinase-2 for Akt in human neutrophils. J Biol Chem 276: 3517–3523. 11042204 10.1074/jbc.M005953200

[54] Hamilton JA, Byrne R, Whitty G (2000) Particulate adjuvants can induce macrophage survival, DNA synthesis, and a synergistic proliferative response to GM-CSF and CSF-1. J Leukoc Biol 67: 226–232. 10670584

[55] Hamilton JA (2003) Nondisposable materials, chronic inflammation, and adjuvant action. J Leukoc Biol 73: 702–712. 12773502 10.1189/jlb.0103037

[56] Hamilton JA (1993) Rheumatoid arthritis: opposing actions of haemopoietic growth factors and slow-acting anti-rheumatic drugs. Lancet 342: 536–539. 8102674 10.1016/0140-6736(93)91653-4

[57] Wang Y, Roche O, Yan MS, Finak G, Evans AJ, et al (2009) Regulation of endocytosis via the oxygen-sensing pathway. Nat Med 15: 319–324. 19252501 10.1038/nm.1922

[58] Mangan DF, Welch GR, Wahl SM (1991) Lipopolysaccharide, tumor necrosis factor-alpha, and IL-1 beta prevent programmed cell death (apoptosis) in human peripheral blood monocytes. J Immunol 146: 1541–1546. 1993844

[59] Nizet V, Johnson RS (2009) Interdependence of hypoxic and innate immune responses. Nat Rev Immunol 9: 609–617. 19704417 10.1038/nri2607 PMC4343208

